# Teaching Scottish medical students about global health in partnership with LMIC institutions. Does it change their views on volunteering in LMIC settings?

**DOI:** 10.1186/s12909-024-05034-9

**Published:** 2024-01-16

**Authors:** Lesley Crichton, Katy Daniels, Neil Merrylees, Marie-Francoise Mukanyangezi, Hazel Mumphansha Sonkwe, Richard Nduwayezu, Emma Thomson

**Affiliations:** 1 School of Medicine, University of Dundee, Dundee, Scotland; 2 University of Rwanda, Kigali, Rwanda; 3 University of Zambia, Lusaka, Zambia; 4 Kamuzu University of Health Sciences, Blantyre, Malawi

**Keywords:** Electives, LMIC, Decolonisation, Curriculum, Global health

## Abstract

**Context:**

An elective placement is a core part of most United Kingdom (UK) medical degrees, and a significant proportion of students choose to pursue their elective in low- and middle-income countries (LMIC). There is a risk that students are ill-prepared for some of the ethical challenges that they will face during these placements, and that they have little appreciation for some of the negative effects that their placement can have on the host healthcare system. This study sought to address some of these negative consequences by exploring the preparation of medical students for these experiences, and the effect of including the LMIC perspective in preparation materials.

**Methods:**

This qualitative study used thematic analysis to explore the attitudes of final year medical students at a Scottish medical school to international volunteering, after completing a module on global health. This module was designed and delivered in partnership with academics from Malawi, Rwanda and Zambia, thus incorporating a strong LMIC perspective.

**Findings:**

This study demonstrated the ability of a global health module with a strong LMIC perspective to influence the attitudes of final year medical students in the following ways: 1) Challenging assumptions around international volunteering and, in particular, around some of the negative effects of international volunteering that had not previously been considered. 2) Changing future practice around international volunteering.

**Implications:**

This study provides good evidence that having a strong LMIC voice in preparation materials for medical students embarking on LMIC electives has the ability to increase awareness of some of the potential harms, and to positively influence how they plan to have discussions around and approach such experiences in the future.

## Background

An elective is part of the curriculum where clinical healthcare students have the flexibility to choose the focus and location of a clinical placement, which is often abroad. Electives are a component of curricula at most medical schools in high income countries (HICs), and are often the first time that students are exposed to health systems abroad and in low- and middle-income countries (LMIC) [1]. There is a risk that students are ill-prepared for the circumstances they will encounter on an elective in a low-resourced setting such as a LMIC, and they may have little awareness of potential negative consequences for the local healthcare system. It is essential, therefore, that we strive to mitigate these risks by dedicating further research to the preparation of medical students for this experience, to subsequently reduce the risk to the host healthcare system as well as to build a lifelong understanding of global health needs.

It is estimated that 40% of UK medical students use their elective to travel to a low-resource setting [1] for a clinical placement, which equates to approximately 4000 per year.

The potential harms of such healthcare placements to the host countries and their healthcare systems, particularly by those from HIC to LMIC, are well described in the literature [2–4]. Given the popularity of LMIC electives at such an early stage in their healthcare career, some of these harms could be perpetuated by these students.

There is little evidence that medical students are aware of potential harms, and few examples exist of them being taught about these harms during their medical training and before they are encouraged to pursue such an elective [5, 6]. Discourse is similarly sparse about the way we teach medical students about global health, although there is some evidence that students who experience global health during their training are more likely to choose to work in settings which concentrate on the underserved [7, 8]. In particular, the opportunity afforded by global health teaching to shape the attitudes of students around what and how they could contribute to health in LMIC, and the effect of that approach to the country’s staff and health system, has not been widely discussed, despite there being some suggestions that those participating in LMIC volunteering can often return to work in a similar setting [2]. This gap in the discourse is present despite general acknowledgement in the literature that the ethical issues around LMIC electives exist [9, 10].

This study aims to address part of this gap in the literature, by exploring how attitudes to international volunteering amongst students are influenced by discussing international volunteering from the LMIC perspective, during a wider global health learning experience at a Scottish medical school. The teaching is delivered through an online module, developed and delivered in partnership with LMIC academics from the Universities of Rwanda and Zambia and Kamuzu University of Health Sciences, Malawi, thus providing the perspective of both HIC and LMIC medical professionals.

### Medical school electives to LMIC

Medical student electives to LMIC are popular, and confer clear benefits to the outgoing students [6, 11–13]. Motivations for HIC students and trainee doctors travelling to LMIC include the pursuit of clinical skills and knowledge, alongside altruism, which is a motivation shared by non-medical volunteers [3, 14–16]. The benefits to the host have been less well studied, however, and there is evidence that these placements can cause a drain on the local teaching and clinical resource, competition with local students, unrealistic expectations, and a lack of cultural competence [2, 3, 9, 17]. There are some recommendations and guidelines for minimising harm, suggesting that good pre-departure preparation and short-term visits in the context of a wider long-term partnership may be beneficial [1, 18, 19]. In all areas, there is less in the literature about the LMIC perspective than reflecting HIC views.

### Global health teaching in medical school curricula

Despite calls from students and academics [20–23], global health remains a topic that is not core or compulsory in most UK medical school curricula, and there is little literature about what GH is taught in LMICs. Some innovative and creative teaching models exist [24–26]. However, there are arguments for moving towards a transformative model, and away from the narrative that global health is “tropical medicine” or health in “other places”. This includes moving towards including international collaborators in the design, creation and delivery of such material [5, 27–29].

### The decolonise movement and global health

Much of what we recognise now as global health was created as an enabler of European colonialism of large parts of the rest of the world [30]. HIC institutions that run medical student electives must acknowledge, consider and mitigate the potential for international volunteering to perpetuate colonial narratives [31]. The move to decolonise global health education is well underway but far from complete [32–35]. Much of this movement is driven by students themselves [36–38], and there are a small number of examples of student-led concern about the colonial overtones of LMIC electives in the literature [38, 39]. There is also little on the topic written with a LMIC perspective. The literature that does exist strongly suggests that equitable partnerships, and a strong voice of those with lived experience of the global health topics being discussed are incorporated into curriculum design and delivery [28, 40–42].

## Methods

We completed a qualitative evaluation to understand the impact the Global Health Perspectives module has on HIC students’ attitudes to volunteering in LMIC.

This study solely examines the attitudes of final year students at the Scottish medical school who completed the module, although it was also completed by students from Malawi, Zambia and Rwanda during the study period. These Scottish students had received very little formal global health teaching in previous parts of their medical degree, although a small number of them (not separated) will have completed an intercalated degree in International Health in the preceding year. Of particular relevance to this study, the module contains the following:

- A section on voluntourism including definitions and examples.
- A discussion between a Zambian doctor and a Scottish doctor with LMIC experience around the harms and the benefits of international volunteering.
- Further reading and references to literature evidencing some of the harms associated with international volunteering.

Following completion of the module students write a short (600 words) reflective essay, with the following instructions:

In this reflective piece students should consider what level of knowledge they had and their attitudes prior to starting this module, where they are now, and how this learning will influence them in the future, i.e. past, present, future.

### Data collection

Secondary data, in the form of excerpts from the reflective essays, were used. Students were asked to outline what level of knowledge they had and their attitudes prior to starting the module, where they were now and how this learning would influence them in the future.

Essays from the first eight months of academic year 2021–2022 were identified; a total of 202. Four were removed as no consent was provided. Therefore 198 essays were examined for mentions of either:

- Volunteering.
- International volunteering.
- Voluntourism.
- Students or medical staff working or on placement in LMIC.

**Fig. 1:**
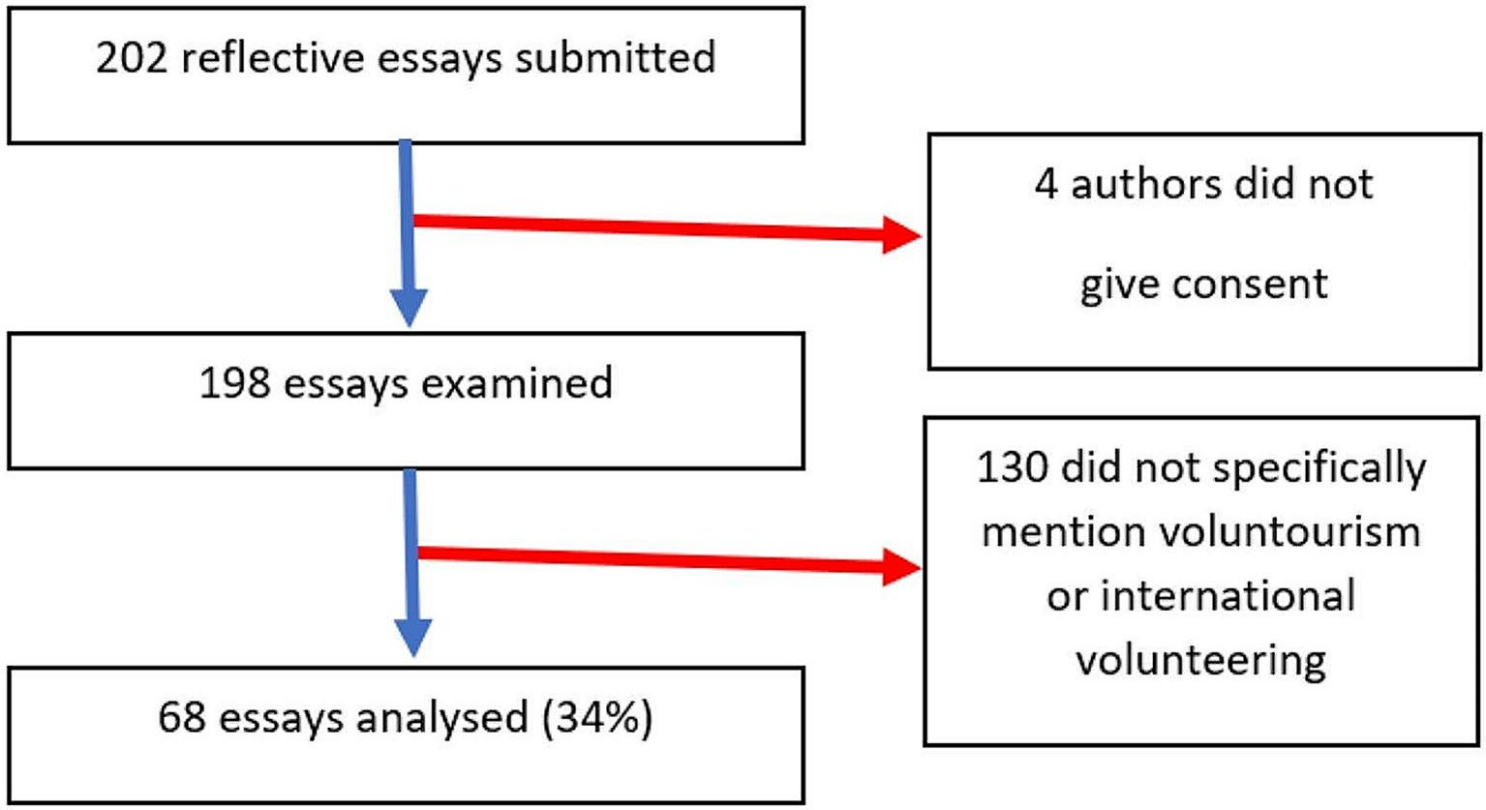
202 essays were submitted. 4 were withdrawn after authors did not give consent. From the remaining 198 essays, 130 did not specifically mention voluntourism or international volunteering, which left 68 essays for the final analysis

Of these, 130 made no mention of the four terms, and were removed from the dataset, leaving 68 essays (34%) as the final sample. Excerpts of the essays discussing the four terms (above) were then selected. This process is illustrated in Fig. 1:

### Data analysis

We undertook thematic analysis, using an inductive approach [43], deriving themes from the data rather than from a pre-existing framework or the investigator’s own interests or beliefs [44, 45]. We took this approach for two reasons: firstly, because there was not enough existing data available in the literature in order to construct a framework with which to start, and most importantly, in order for us to be able to elicit unanticipated views and student-generated themes that had previously not been considered.

We allocated each essay a reference number from E1-68, and these were subsequently analysed using NVivo software, where initial codes were generated and applied [46]. We carried out coding using a constructivist approach, where data were used to construct themes rather than starting the process with a thematic framework in mind. These codes were then grouped into themes. The first author, who has previous experience of the process, was the one who primarily carried out the analysis, with themes being checked by the whole group. Any disagreements were addressed at online meetings of the group.

## Results

**Fig. 2:**
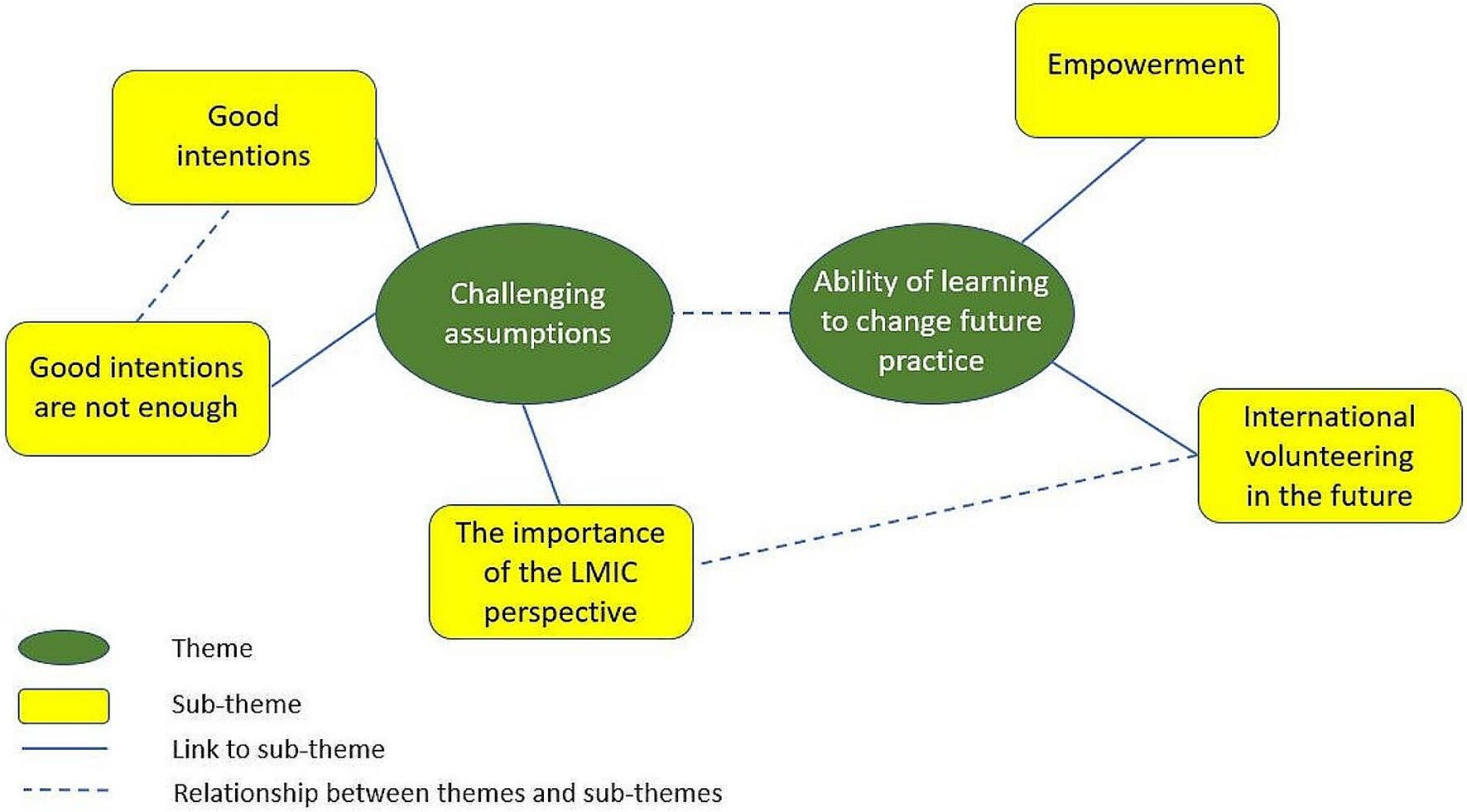
Shows the two major themes, and five interlinked sub-themes constructed from the reflective essay data

We constructed two major themes and five interconnected subthemes from the reflective essay data (Fig. 2):

### Major theme 1

Challenging Assumptions - The fact that international healthcare volunteering was well-intentioned, but that the potential harms had not been previously considered. (Subthemes 1–3)

### Major theme 2

The ability of this learning to change future practice– practically in terms of intended trips, but also on the ability to think critically and to challenge others’ beliefs in the future. (Subthemes 4 and 5)

### First major theme: challenging assumptions

#### Sub-theme 1: good intentions

The first theme was the recognition that international health volunteering is largely altruistic (15 students):

*“I think that previously I was guilty of thinking students and healthcare professionals from the UK were ‘helping’ communities in low- and middle-income countries by undertaking work there, while also gaining valuable knowledge and experience themselves” (E 52)*

Good intentions are key– this is a feature of international volunteering which is prevalent and is so important to include in the discussions around the ethics of the practice.

*“students often go with good intentions and just want to help”. (E31)*

#### Sub-theme 2: good intentions are not enough

The second part was the lack of appreciation of the potential negative aspects of voluntourism. This theme was characterised by several students expressing particularly strong feelings such as “shame”, “embarrassment” and “horror”, which served as a stark reminder that these discussions can often be emotionally challenging, and that support should be offered to those taking part.

There were some students who were faced with these potential negatives for the first time:

*“I was naïve to this concept” (E28)*.

*“I had not fully appreciated the negative effects of [voluntourism].” (E54)*.

For some others, there was the ability to give a vocabulary to unease they felt about past experiences:

*“I have learned that I took part in voluntourism when I was about 16/17-years-old” (E42)*.

And to revisit and reflect on their feelings about past experiences:

*“Having considered my own impact working in these regions, I have many a time considered whether I had a net positive or negative impact on the settings I worked within.” (E63)*.

#### Sub-theme 3: the importance of the LMIC perspective

For 13 students, the stories from LMIC staff were a powerful tool:

*“learning about the thoughts and feelings from the host countries taught me the negative impacts we can have on them” (E56)*.

### Second major theme: the ability of the learning to change future practice

The second thematic group was the power of this learning to change students’ future plans and practice around international volunteering, with 32 students suggesting that their future practice would change as a result.

#### Sub-theme 4: empowerment

There was a feeling of empowerment (3 students) to discuss this area and to challenge others

*“I found it incredibly useful to hear someone articulate some of the thoughts and feeling that I had had on the topic in a way that might allow me to openly questions such attitudes in the future.” (E6)*.

*“I now feel better equipped with reliable sources and better knowledge to discuss this issue with peers.” (E35)*.

#### Sub-theme 5: international volunteering in the future

While 18 students mentioned that they hoped to participate in international volunteering in the future, there were examples of the ways that these students would change their future practice, including in preparation and on the trip itself:

There was discussion around good preparation for international volunteering:

*“I would learn as much as I can about local culture, languages spoken and the challenges faced by communities before travelling (…) in order to minimise the strain I place on the health care system and members of staff.” (E49)*.

25 students wrote about cultural awareness and respecting different ways of working:

*“Some of the main issues that can arise are cultural insensitivities and naivety of individuals to cultural and structural constraints of the healthcare system they are placed within. These placements are typically time-limited, and the rapid turnover of either students disrupts partnerships and can lead to a superficial understanding of the culture” (E1)*.

Two students even came to the conclusion that their plans should be cancelled or postponed:

*“Considering a medical elective in a LMIC, I know I would be more of a burden to the host hospital and that undertaking a medical elective in a LMIC would serve my needs more than the needs of the host hospital, which is not a truly equitable exchange.” (E51)*.

This suggests that incorporating the LMIC perspective into teaching material can have a profound effect on behaviour and attitudes. These examples demonstrate that the ability to think critically about international volunteering has brought students to their own conclusions about their own position to the point where they might even alter future plans to volunteer.

## Discussion

### Key findings from this study

As exporters of large numbers of medical students to LMIC to embark on healthcare volunteering, it is incumbent upon HIC medical schools that they equip medical students to be able to pursue these trips in a way that is ethical and does as little harm to the host communities and healthcare systems as possible. However, there is little information in the literature about the potential areas of harm, and how medical schools should prepare medical students in such a way that reduces this risk.

Our study demonstrates that having a strong LMIC perspective in teaching material is a powerful way to ensure students are alive to some of the risks of LMIC volunteering to the host countries, and that they are able to think critically about their past and future plans, and feel empowered to discuss and debate these with others. Having the LMIC perspective enables students to think past their good intentions and consider the unintended harms that international volunteering can cause.

In addition to these primary findings, this study also found that being confronted by some of these risks and harms of international volunteering elicited some strong emotions and precipitated some frank self-reflection, which suggests that these essential conversations must be approached sensitively and with care. It is the opinion of the authors, however, that these conversations must take place, however difficult they are, to minimise harm that may unwittingly be caused through ignorance, however well-intentioned.

### The findings from this study in context

There are already examples and recommendations in the literature of ways to develop undergraduate medical GH teaching and electives preparation to bring in the LMIC perspective and to develop material in partnership with academics and institutions in LMIC [5, 28, 47]. There is little, however, about the effect of this methodology on the attitudes and beliefs of students around international volunteering, which is the gap in the literature that this particular study has sought to address.

Secondly, regarding best practice guidelines and advice for ethical conduct during international volunteering for medical students; again, there are good examples of these in the literature [18, 48, 49]. However, there is little discussion around whether student attitudes are in line with these recommendations and whether they are followed. Again, the authors sought to address this deficit by exploring whether students would aim to change their future practice around international volunteering in light of their new learning. This study demonstrates that a GH module with a strong LMIC perspective has the ability to push medical students to reach some of the conclusions from these guidelines themselves.

Thirdly, there is an active discourse in contemporary literature about decolonising global health and ensuring the power structures, in terms of literature, research, organisations and much more are recognised and dismantled [30, 34, 40, 41]. This wider conversation is beyond the scope of this study, but, given that the export of medical students to participate in volunteering in LMIC sits within the scope of the decolonising conversation, we believe it is important to acknowledge that electives must also be decolonised. This study demonstrates that by weaving the LMIC perspective into teaching material, students are able to come to their own conclusion that international volunteering can perpetuate colonial attitudes, inequities and power imbalances.

Therefore, designing material in partnership with LMIC is key. The earlier this is done the better, so that LMIC perspectives are woven into all conversations around global health in medical school, and students are more likely to engage in LMIC volunteering in a way that does less harm and is more equitable.

### Limitations

This study took place using data from one medical school year group only, during the Covid-19 pandemic.

The Scottish University authors held a position of authority over the students whose essays were used as secondary data for this study. This could potentially affect the ability of students to withdraw consent if they were not comfortable with participating in the study.

Students were asked to discuss learning that was important to them rather than specifically asking students to express their attitudes around international volunteering, which could be viewed as a strength of the study. The fact that around a third of them chose international volunteering as a topic to discuss suggests that the GHP module was successful in its aim of challenging students’ attitudes in this particular area.

### Recommendations for HIC medical schools

- All HIC medical schools should have core learning in global health for all students.
- This learning should be early enough in the curriculum for students to make informed decisions about their elective - for some in our study this came too late.
- GH learning must contain content on the ethics of international volunteering.
- Content must be developed and delivered with a strong perspective of LMIC healthcare providers/placement hosts.

## Conclusions

The findings of this study demonstrate that good intentions are not enough. Significant numbers of HIC medical students embark on electives to LMIC each year, and motives are largely altruistic. In our study, it had simply not occurred to students that there could be any harms associated with these trips, and there were often strong feelings associated with this realisation.

A strong LMIC host perspective in the learning material, deemed the intervention in this study, was credited as a powerful way to demonstrate some of these harms. The consequence being students feeling empowered to critically think about and discuss this topic with others, reflect on past international volunteering experiences critically, and to examine, better prepare for and, in some cases, change future international volunteering plans in light of their new learning.

This study concludes that giving voice to the LMIC hosts and people directly affected by incoming HIC volunteers is essential to minimise the harms and capitalise on the many benefits that can potentially result from these trips.

## Data Availability

The datasets used and/or analysed during the current study are available from the corresponding author on reasonable request.

## List of abbreviations

GH: Global health
GHP: Global Health Perspectives (module)
HIC: High-income countries
IHE: International healthcare elective
LMIC: Low- and middle-income countries
NHS: National Health Service (UK)
